# 5-Fluoro-*N*′-[(*E*)-4-methoxy­benzyl­idene]-3-phenyl-1*H*-indole-2-carbohydrazide

**DOI:** 10.1107/S1600536810009098

**Published:** 2010-03-13

**Authors:** Mehmet Akkurt, Ísmail Çelik, Gökçe Cihan, Gültaze Çapan, Orhan Büyükgüngör

**Affiliations:** a Department of Physics, Faculty of Arts and Sciences, Erciyes University, 38039 Kayseri, Turkey; b Department of Physics, Faculty of Arts and Sciences, Cumhuriyet University, 58140 Sivas, Turkey; c Department of Pharmaceutical Chemistry, Faculty of Pharmacy, University of Istanbul, 34116 Beyazıt, Istanbul, Turkey; d Department of Physics, Faculty of Arts and Sciences, Ondokuz Mayıs University, 55139 Samsun, Turkey

## Abstract

In the title mol­ecule, C_23_H_18_FN_3_O_2_, the mean plane of the indole system forms dihedral angles of 44.23 (8) and 14.54 (7)°, respectively, with the phenyl and benzene rings. In the crystal, inter­molecular N—H⋯O hydrogen bonds link mol­ecules into two-layer ribbons extended along the *b* axis. The crystal packing also exhibits weak inter­molecular C—H⋯O, C—H⋯F and C—H⋯π inter­actions.

## Related literature

For the synthesis and characterization of related indole derivatives, see: Akkurt *et al.* (2009 ▶); Güzel *et al.* (2006 ▶); Kaynak *et al.* (2005 ▶). For typical values of bond lengths in organic compounds, see: Allen *et al.* (1987 ▶).
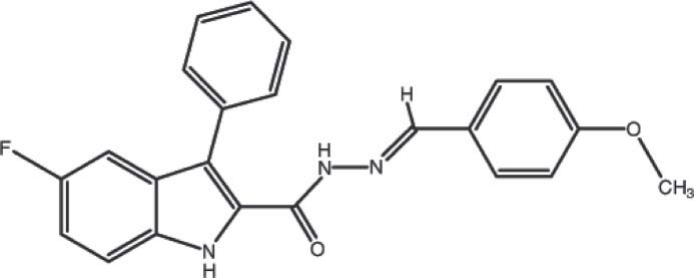


## Experimental

### 

#### Crystal data

C_23_H_18_FN_3_O_2_
*M* *_r_* = 387.40
Monoclinic, 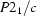

*a* = 19.5709 (11) Å
*b* = 5.1546 (2) Å
*c* = 24.3584 (14) Å
β = 127.686 (3)°
*V* = 1944.63 (19) Å^3^
*Z* = 4
Mo *K*α radiation
μ = 0.09 mm^−1^
*T* = 296 K
0.60 × 0.38 × 0.07 mm

#### Data collection

Stoe IPDS2 diffractometer
Absorption correction: integration (*X-RED32*; Stoe & Cie, 2002 ▶) *T*_min_ = 0.958, *T*_max_ = 0.994
23274 measured reflections
4053 independent reflections
2900 reflections with *I* > 2σ(*I*)
*R*_int_ = 0.038

#### Refinement

*R*[*F* ^2^ > 2σ(*F* ^2^)] = 0.038
*wR*(*F* ^2^) = 0.096
*S* = 1.03
4053 reflections
264 parameters
H-atom parameters constrained
Δρ_max_ = 0.12 e Å^−3^
Δρ_min_ = −0.13 e Å^−3^

### 

Data collection: *X-AREA* (Stoe & Cie, 2002 ▶); cell refinement: *X-AREA*; data reduction: *X-RED32* (Stoe & Cie, 2002 ▶); program(s) used to solve structure: *SIR97* (Altomare *et al.*, 1999 ▶); program(s) used to refine structure: *SHELXL97* (Sheldrick, 2008 ▶); molecular graphics: *ORTEP-3* (Farrugia, 1997 ▶); software used to prepare material for publication: *WinGX* (Farrugia, 1999 ▶).

## Supplementary Material

Crystal structure: contains datablocks global, I. DOI: 10.1107/S1600536810009098/cv2701sup1.cif

Structure factors: contains datablocks I. DOI: 10.1107/S1600536810009098/cv2701Isup2.hkl

Additional supplementary materials:  crystallographic information; 3D view; checkCIF report

## Figures and Tables

**Table 1:** Hydrogen-bond geometry (Å, °) *Cg*1 is the centroid of the N1/C1/C6–C8 ring.

*D*—H⋯*A*	*D*—H	H⋯*A*	*D*⋯*A*	*D*—H⋯*A*
N1—H1⋯O1^i^	0.86	2.03	2.8573 (19)	162
N2—H2*A*⋯O1^ii^	0.86	2.32	3.0373 (14)	141
C3—H3⋯F1^iii^	0.93	2.54	3.473 (2)	177
C16—H16⋯O1^ii^	0.93	2.50	3.1939 (18)	131
C10—H10⋯*Cg*1^ii^	0.93	2.85	3.3736 (17)	117

Symmetry codes: (i) 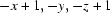; (ii) 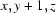; (iii) 
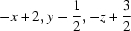
.

**Fig. 1.**
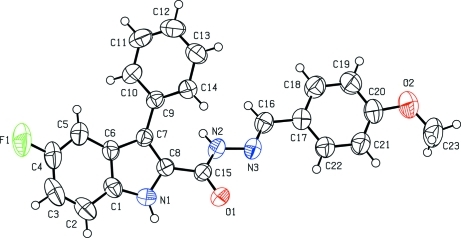
View of the title molecule showing the atomic numbering and 50% probability displacement ellipsoids.

## supplementary crystallographic information

### Comment

In the framework of our ongoing project directed towards the design, synthesis and characterization of bioactive indole derivatives (Akkurt *et al.*, 2009; Güzel *et al.*, 2006; Kaynak *et al.*, 2005), we report here the synthesis and crystal structure of the title compound.

In the title molecule (Fig. 1), bond lengths and bond angles are all within expected values (Allen *et al.*, 1987). The nine-membered indole ring [a maximum deviation from the mean plane of 0.039 (2) Å for C6] makes dihedral angles of 44.23 (8)° and 14.54 (7)°, respectively, with the phenyl ring (C9–C14) and benzene ring (C17–C22). The (C9–C14) and (C17–C22) rings form a dihedral angle of 36.87 (9)°.

In the crystal structure, intermolecular N—H···O hydrogen bonds link molecules into two-layer ribbons extended along *b* axis. The crystal packing exhibits also weak intermolecular C—H···O, C—H···F and C—H···π interactions (Table 1).

### Experimental

A mixture of 5-fluoro-3-phenyl-1*H*-indole-2-carbohydrazide (0.005 mol), 4-methoxy benzaldehyde (0.006 mol) and 15 ml of absolute ethanol was heated on a water bath for 4 h. The crude product obtained on cooling was filtered and purified by recrystallization from ethanol/chloroform (1/1). [Yield: 93.8 %, m.p.: 493-493 K]. IR (KBr) ν = 3247 (N—H), 1635 (C=O), 1622 (C=N) cm^-1^; ^1^H-NMR (DMSO-d_6_, 500 MHz) δ = 3.78 (3*H*, s, 4-OCH_3_), 6.99 (2*H*, d*, J=6.8 Hz, H3,H5-benzylidene), 7.14 (1*H*, t*, J=8.8 Hz, H6-indole), 7.31-7.52 (7*H*, m, H4, H7, 3-C_6_H_5_-indole), 7.62 (2*H*, d*, J=5.9 Hz, H2, H6-benzylidene), 8.02 (1*H*, s, N=CH), 11.23 (1*H*, s, CONH), 12.02 (1*H*, s, NH-indole) ppm ( * = broad). Analysis calculated for C_23_H_18_FN_3_O_2_ : C 71.31, H 4.68, N 10.85 %. Found : C 71.22, H 3.94, N 10.82 %.

### Refinement

H atoms were placed in idealized positions with N—H = 0.86 Å and C—H = 0.93 - 0.96 Å, and constrained to ride on their parent atoms with *U*_iso_(H) = 1.2 or 1.5*U*_eq_(C, N).

### Crystal data

**Table:**

C_23_H_18_FN_3_O_2_	*F*(000) = 808
*M**_r_* = 387.40	*D*_x_ = 1.323 Mg m^−^^3^
Monoclinic, *P*2_1_/*c*	Mo *K*α radiation, λ = 0.71073 Å
Hall symbol: -P 2ybc	Cell parameters from 8633 reflections
*a* = 19.5709 (11) Å	θ = 1.8–27.3°
*b* = 5.1546 (2) Å	µ = 0.09 mm^−^^1^
*c* = 24.3584 (14) Å	*T* = 296 K
β = 127.686 (3)°	Plate, colourless
*V* = 1944.63 (19) Å^3^	0.60 × 0.38 × 0.07 mm
*Z* = 4	

### Data collection

**Table:**

Stoe IPDS2 diffractometer	4053 independent reflections
Radiation source: sealed X-ray tube, 12 x 0.4 mm long-fine focus	2900 reflections with *I* > 2σ(*I*)
plane graphite	*R*_int_ = 0.038
Detector resolution: 6.67 pixels mm^-1^	θ_max_ = 26.5°, θ_min_ = 1.3°
ω scans	*h* = −24→24
Absorption correction: integration (*X-RED32*; Stoe & Cie, 2002)	*k* = −6→6
*T*_min_ = 0.958, *T*_max_ = 0.994	*l* = −30→30
23274 measured reflections	

### Refinement

**Table:**

Refinement on *F*^2^	Secondary atom site location: difference Fourier map
Least-squares matrix: full	Hydrogen site location: inferred from neighbouring sites
*R*[*F*^2^ > 2σ(*F*^2^)] = 0.038	H-atom parameters constrained
*wR*(*F*^2^) = 0.096	*w* = 1/[σ^2^(*F*_o_^2^) + (0.0532*P*)^2^] where *P* = (*F*_o_^2^ + 2*F*_c_^2^)/3
*S* = 1.03	(Δ/σ)_max_ < 0.001
4053 reflections	Δρ_max_ = 0.12 e Å^−^^3^
264 parameters	Δρ_min_ = −0.12 e Å^−^^3^
0 restraints	Extinction correction: *SHELXL97* (Sheldrick, 2008), Fc^*^=kFc[1+0.001xFc^2^λ^3^/sin(2θ)]^-1/4^
Primary atom site location: structure-invariant direct methods	Extinction coefficient: 0.0123 (12)

### Special details

**Table:**

Geometry. Bond distances, angles etc. have been calculated using the rounded fractional coordinates. All su's are estimated from the variances of the (full) variance-covariance matrix. The cell esds are taken into account in the estimation of distances, angles and torsion angles
Refinement. Refinement on *F*^2^ for ALL reflections except those flagged by the user for potential systematic errors. Weighted *R*-factors wR and all goodnesses of fit *S* are based on *F*^2^, conventional *R*-factors *R* are based on *F*, with *F* set to zero for negative *F*^2^. The observed criterion of *F*^2^ > σ(*F*^2^) is used only for calculating -*R*-factor-obs etc. and is not relevant to the choice of reflections for refinement. *R*-factors based on *F*^2^ are statistically about twice as large as those based on *F*, and *R*-factors based on ALL data will be even larger.

### Fractional atomic coordinates and isotropic or equivalent isotropic displacement parameters (Å^2^)

**Table:**

	*x*	*y*	*z*	*U*_iso_*/*U*_eq_	
F1	0.96473 (6)	0.3955 (3)	0.67120 (6)	0.1153 (5)	
O1	0.46022 (6)	0.08712 (18)	0.41796 (5)	0.0624 (3)	
O2	−0.00747 (7)	0.7292 (3)	0.05087 (6)	0.0898 (4)	
N1	0.62743 (7)	0.1715 (2)	0.53758 (5)	0.0565 (4)	
N2	0.45250 (7)	0.5105 (2)	0.38898 (6)	0.0532 (4)	
N3	0.36665 (7)	0.4917 (2)	0.33221 (6)	0.0553 (4)	
C1	0.71455 (9)	0.2113 (3)	0.57970 (7)	0.0560 (4)	
C2	0.77885 (11)	0.0917 (3)	0.64212 (8)	0.0747 (6)	
C3	0.86269 (11)	0.1589 (4)	0.67208 (8)	0.0867 (7)	
C4	0.87981 (9)	0.3381 (4)	0.63954 (8)	0.0778 (6)	
C5	0.81907 (9)	0.4567 (3)	0.57871 (7)	0.0637 (5)	
C6	0.73246 (8)	0.3953 (3)	0.54742 (6)	0.0505 (4)	
C7	0.65172 (8)	0.4668 (2)	0.48304 (6)	0.0464 (4)	
C8	0.58921 (8)	0.3256 (2)	0.47996 (6)	0.0480 (4)	
C9	0.64238 (8)	0.6238 (2)	0.42835 (6)	0.0465 (4)	
C10	0.69245 (9)	0.8454 (2)	0.44513 (8)	0.0567 (4)	
C11	0.68968 (10)	0.9792 (3)	0.39482 (10)	0.0702 (6)	
C12	0.63650 (11)	0.8975 (3)	0.32699 (10)	0.0775 (7)	
C13	0.58566 (10)	0.6816 (3)	0.30908 (8)	0.0686 (5)	
C14	0.58882 (8)	0.5452 (3)	0.35932 (7)	0.0544 (4)	
C15	0.49563 (8)	0.2983 (3)	0.42673 (6)	0.0493 (4)	
C16	0.33397 (9)	0.6934 (3)	0.29425 (7)	0.0565 (5)	
C17	0.24446 (8)	0.6999 (3)	0.23225 (7)	0.0546 (4)	
C18	0.21612 (10)	0.8892 (3)	0.18233 (9)	0.0710 (6)	
C19	0.13225 (11)	0.8934 (3)	0.12238 (9)	0.0781 (6)	
C20	0.07431 (9)	0.7099 (3)	0.11180 (8)	0.0672 (5)	
C21	0.10054 (10)	0.5219 (3)	0.16103 (8)	0.0712 (5)	
C22	0.18461 (9)	0.5177 (3)	0.22030 (8)	0.0665 (5)	
C23	−0.06706 (11)	0.5297 (4)	0.03519 (11)	0.0994 (8)	
H1	0.60070	0.06620	0.54590	0.0680*	
H2	0.76550	−0.02890	0.66270	0.0900*	
H2A	0.47840	0.65790	0.40020	0.0640*	
H3	0.90770	0.08460	0.71400	0.1040*	
H5	0.83390	0.57360	0.55850	0.0760*	
H10	0.72810	0.90370	0.49080	0.0680*	
H11	0.72400	1.12550	0.40680	0.0840*	
H12	0.63490	0.98820	0.29320	0.0930*	
H13	0.54910	0.62740	0.26310	0.0820*	
H14	0.55460	0.39840	0.34680	0.0650*	
H16	0.36820	0.84010	0.30650	0.0680*	
H18	0.25450	1.01630	0.18940	0.0850*	
H19	0.11480	1.02040	0.08910	0.0940*	
H21	0.06150	0.39790	0.15420	0.0850*	
H22	0.20180	0.38940	0.25320	0.0800*	
H23A	−0.04400	0.36500	0.03540	0.1490*	
H23B	−0.12070	0.56020	−0.00980	0.1490*	
H23C	−0.07670	0.52920	0.06940	0.1490*	

### Atomic displacement parameters (Å^2^)

**Table:**

	*U*^11^	*U*^22^	*U*^33^	*U*^12^	*U*^13^	*U*^23^
F1	0.0444 (5)	0.1659 (11)	0.0877 (7)	0.0028 (6)	0.0158 (5)	−0.0115 (7)
O1	0.0607 (6)	0.0602 (6)	0.0590 (6)	−0.0204 (5)	0.0328 (5)	−0.0045 (4)
O2	0.0559 (7)	0.1070 (9)	0.0738 (7)	0.0105 (6)	0.0229 (6)	0.0101 (7)
N1	0.0587 (7)	0.0636 (7)	0.0512 (6)	−0.0052 (5)	0.0357 (6)	0.0048 (5)
N2	0.0418 (6)	0.0510 (6)	0.0596 (7)	−0.0069 (5)	0.0273 (5)	−0.0058 (5)
N3	0.0421 (6)	0.0623 (7)	0.0557 (6)	−0.0046 (5)	0.0270 (5)	−0.0076 (5)
C1	0.0562 (8)	0.0663 (8)	0.0436 (7)	0.0036 (7)	0.0296 (7)	−0.0009 (6)
C2	0.0781 (11)	0.0916 (11)	0.0505 (8)	0.0176 (9)	0.0373 (8)	0.0118 (8)
C3	0.0666 (11)	0.1203 (15)	0.0461 (8)	0.0268 (10)	0.0205 (8)	0.0064 (9)
C4	0.0457 (8)	0.1054 (13)	0.0574 (9)	0.0035 (8)	0.0187 (8)	−0.0120 (9)
C5	0.0477 (8)	0.0754 (9)	0.0565 (8)	−0.0037 (7)	0.0260 (7)	−0.0089 (7)
C6	0.0462 (7)	0.0571 (7)	0.0434 (7)	−0.0029 (6)	0.0249 (6)	−0.0067 (6)
C7	0.0442 (7)	0.0484 (7)	0.0450 (7)	−0.0053 (5)	0.0264 (6)	−0.0050 (5)
C8	0.0488 (7)	0.0504 (7)	0.0446 (7)	−0.0046 (6)	0.0285 (6)	−0.0021 (6)
C9	0.0426 (6)	0.0467 (6)	0.0531 (7)	0.0014 (5)	0.0307 (6)	0.0012 (5)
C10	0.0521 (7)	0.0503 (7)	0.0696 (9)	−0.0029 (6)	0.0382 (7)	−0.0014 (6)
C11	0.0626 (9)	0.0576 (8)	0.0977 (13)	0.0018 (7)	0.0528 (10)	0.0177 (8)
C12	0.0767 (11)	0.0853 (11)	0.0863 (12)	0.0158 (9)	0.0580 (10)	0.0338 (10)
C13	0.0667 (9)	0.0824 (10)	0.0571 (8)	0.0094 (8)	0.0380 (8)	0.0131 (8)
C14	0.0503 (7)	0.0584 (8)	0.0519 (8)	0.0003 (6)	0.0299 (7)	0.0038 (6)
C15	0.0494 (7)	0.0548 (7)	0.0472 (7)	−0.0108 (6)	0.0314 (6)	−0.0074 (6)
C16	0.0528 (8)	0.0566 (8)	0.0606 (8)	−0.0048 (6)	0.0349 (7)	−0.0101 (7)
C17	0.0506 (7)	0.0562 (7)	0.0556 (8)	0.0026 (6)	0.0318 (7)	−0.0065 (6)
C18	0.0625 (9)	0.0616 (9)	0.0798 (11)	−0.0024 (7)	0.0388 (9)	0.0014 (8)
C19	0.0693 (10)	0.0719 (10)	0.0750 (10)	0.0081 (8)	0.0349 (9)	0.0150 (8)
C20	0.0513 (8)	0.0749 (10)	0.0608 (9)	0.0086 (7)	0.0268 (7)	−0.0027 (8)
C21	0.0516 (8)	0.0766 (10)	0.0718 (10)	−0.0047 (7)	0.0308 (8)	0.0023 (8)
C22	0.0538 (8)	0.0694 (9)	0.0637 (9)	−0.0012 (7)	0.0295 (7)	0.0068 (7)
C23	0.0472 (9)	0.1322 (17)	0.0881 (13)	−0.0010 (10)	0.0257 (9)	−0.0051 (12)

### Geometric parameters (Å, °)

**Table:**

F1—C4	1.370 (2)	C12—C13	1.374 (3)
O1—C15	1.2369 (19)	C13—C14	1.380 (2)
O2—C20	1.367 (2)	C16—C17	1.455 (2)
O2—C23	1.421 (3)	C17—C22	1.386 (3)
N1—C1	1.365 (2)	C17—C18	1.383 (2)
N1—C8	1.3699 (15)	C18—C19	1.377 (3)
N2—N3	1.3798 (19)	C19—C20	1.375 (3)
N2—C15	1.3437 (18)	C20—C21	1.374 (2)
N3—C16	1.2735 (18)	C21—C22	1.374 (3)
N1—H1	0.8600	C2—H2	0.9300
N2—H2A	0.8600	C3—H3	0.9300
C1—C2	1.391 (2)	C5—H5	0.9300
C1—C6	1.406 (2)	C10—H10	0.9300
C2—C3	1.371 (3)	C11—H11	0.9300
C3—C4	1.385 (3)	C12—H12	0.9300
C4—C5	1.352 (2)	C13—H13	0.9300
C5—C6	1.405 (3)	C14—H14	0.9300
C6—C7	1.4360 (19)	C16—H16	0.9300
C7—C8	1.386 (2)	C18—H18	0.9300
C7—C9	1.4715 (18)	C19—H19	0.9300
C8—C15	1.467 (2)	C21—H21	0.9300
C9—C14	1.3911 (18)	C22—H22	0.9300
C9—C10	1.3937 (19)	C23—H23A	0.9600
C10—C11	1.378 (3)	C23—H23B	0.9600
C11—C12	1.374 (3)	C23—H23C	0.9600
			
F1···H3^i^	2.5400	C14···H11^iii^	3.0500
F1···H21^ii^	2.6500	C15···H1^iv^	3.0100
O1···N1	2.7803 (16)	C15···H14	2.8500
O1···N2^iii^	3.0373 (14)	C16···H12^ix^	2.7600
O1···N3	2.7182 (15)	C17···H12^ix^	2.9900
O1···C16^iii^	3.1939 (18)	C21···H23C	2.7500
O1···N1^iv^	2.8573 (19)	C21···H23A	2.7300
O1···H2A^iii^	2.3200	C23···H21	2.5100
O1···H16^iii^	2.5000	C23···H5^x^	3.1000
O1···H1	2.6100	H1···O1	2.6100
O1···H1^iv^	2.0300	H1···O1^iv^	2.0300
O2···H23A^v^	2.8700	H1···C15^iv^	3.0100
N1···O1	2.7803 (16)	H2···H22^iv^	2.5400
N1···O1^iv^	2.8573 (19)	H2A···O1^vi^	2.3200
N2···O1^vi^	3.0373 (14)	H2A···C7	2.8600
N2···C9	3.273 (2)	H2A···C9	2.8500
N2···C14	3.167 (2)	H2A···C14	2.9400
N3···O1	2.7182 (15)	H2A···H16	2.1700
N2···H14	2.8100	H3···F1^xi^	2.5400
N3···H22	2.6100	H5···C10	2.8100
C1···C10^iii^	3.572 (2)	H5···H10	2.3900
C2···C22^vii^	3.587 (2)	H5···C23^viii^	3.1000
C2···C16^vii^	3.562 (3)	H5···H23C^viii^	2.6000
C2···C17^vii^	3.526 (3)	H10···C1^vi^	2.8200
C5···C10	3.299 (2)	H10···C5	2.9000
C6···C10^iii^	3.5313 (19)	H10···C6	2.9400
C7···C10^iii^	3.5564 (17)	H10···C6^vi^	2.8600
C9···N2	3.273 (2)	H10···H5	2.3900
C10···C7^vi^	3.5564 (17)	H11···C14^vi^	3.0500
C10···C6^vi^	3.5313 (19)	H11···H23B^viii^	2.5900
C10···C5	3.299 (2)	H12···C16^xii^	2.7600
C10···C1^vi^	3.572 (2)	H12···C17^xii^	2.9900
C11···C14^vi^	3.327 (2)	H13···H14^xii^	2.5700
C14···N2	3.167 (2)	H14···N2	2.8100
C14···C15	3.362 (2)	H14···C8	2.9000
C14···C11^iii^	3.327 (2)	H14···C11^iii^	3.0400
C15···C14	3.362 (2)	H14···C15	2.8500
C16···C2^vii^	3.562 (3)	H14···H13^ix^	2.5700
C16···O1^vi^	3.1939 (18)	H16···O1^vi^	2.5000
C17···C2^vii^	3.526 (3)	H16···H2A	2.1700
C18···C22^vi^	3.525 (2)	H16···H18	2.4800
C19···C21^vi^	3.533 (2)	H18···H16	2.4800
C21···C19^iii^	3.533 (2)	H21···C23	2.5100
C22···C18^iii^	3.525 (2)	H21···H23A	2.3200
C22···C2^vii^	3.587 (2)	H21···H23C	2.2800
C1···H10^iii^	2.8200	H21···F1^xiii^	2.6500
C4···H23C^ii^	3.0000	H22···N3	2.6100
C5···H10	2.9000	H22···H2^iv^	2.5400
C6···H10^iii^	2.8600	H23A···C21	2.7300
C6···H10	2.9400	H23A···H21	2.3200
C7···H2A	2.8600	H23A···O2^v^	2.8700
C8···H14	2.9000	H23B···C11^x^	2.9400
C9···H2A	2.8500	H23B···H11^x^	2.5900
C10···H5	2.8100	H23C···C21	2.7500
C11···H23B^viii^	2.9400	H23C···H21	2.2800
C11···H14^vi^	3.0400	H23C···C4^xiii^	3.0000
C14···H2A	2.9400	H23C···H5^x^	2.6000
			
C20—O2—C23	117.57 (15)	C16—C17—C18	120.67 (16)
C1—N1—C8	109.37 (13)	C17—C18—C19	121.38 (18)
N3—N2—C15	119.95 (11)	C18—C19—C20	119.99 (16)
N2—N3—C16	115.70 (13)	O2—C20—C19	115.94 (15)
C8—N1—H1	125.00	C19—C20—C21	119.76 (17)
C1—N1—H1	125.00	O2—C20—C21	124.30 (17)
N3—N2—H2A	120.00	C20—C21—C22	119.78 (18)
C15—N2—H2A	120.00	C17—C22—C21	121.69 (15)
N1—C1—C2	129.38 (17)	C1—C2—H2	121.00
N1—C1—C6	107.75 (12)	C3—C2—H2	121.00
C2—C1—C6	122.80 (18)	C2—C3—H3	120.00
C1—C2—C3	117.27 (18)	C4—C3—H3	120.00
C2—C3—C4	119.55 (16)	C4—C5—H5	122.00
F1—C4—C5	118.06 (18)	C6—C5—H5	121.00
C3—C4—C5	124.78 (19)	C9—C10—H10	120.00
F1—C4—C3	117.16 (16)	C11—C10—H10	120.00
C4—C5—C6	116.96 (16)	C10—C11—H11	120.00
C5—C6—C7	133.76 (14)	C12—C11—H11	120.00
C1—C6—C5	118.62 (13)	C11—C12—H12	120.00
C1—C6—C7	107.44 (14)	C13—C12—H12	120.00
C8—C7—C9	128.53 (12)	C12—C13—H13	120.00
C6—C7—C9	125.07 (15)	C14—C13—H13	120.00
C6—C7—C8	105.66 (11)	C9—C14—H14	120.00
N1—C8—C15	117.04 (13)	C13—C14—H14	119.00
C7—C8—C15	133.02 (11)	N3—C16—H16	119.00
N1—C8—C7	109.76 (13)	C17—C16—H16	119.00
C7—C9—C10	120.62 (11)	C17—C18—H18	119.00
C7—C9—C14	121.26 (12)	C19—C18—H18	119.00
C10—C9—C14	117.93 (13)	C18—C19—H19	120.00
C9—C10—C11	120.73 (14)	C20—C19—H19	120.00
C10—C11—C12	120.33 (17)	C20—C21—H21	120.00
C11—C12—C13	119.95 (18)	C22—C21—H21	120.00
C12—C13—C14	120.00 (15)	C17—C22—H22	119.00
C9—C14—C13	121.05 (15)	C21—C22—H22	119.00
N2—C15—C8	116.70 (13)	O2—C23—H23A	109.00
O1—C15—N2	123.16 (14)	O2—C23—H23B	109.00
O1—C15—C8	120.14 (13)	O2—C23—H23C	109.00
N3—C16—C17	121.61 (15)	H23A—C23—H23B	109.00
C16—C17—C22	121.94 (14)	H23A—C23—H23C	110.00
C18—C17—C22	117.39 (15)	H23B—C23—H23C	109.00
			
C23—O2—C20—C19	174.44 (18)	C6—C7—C8—N1	−1.05 (15)
C23—O2—C20—C21	−5.2 (3)	C9—C7—C8—C15	−5.4 (2)
C1—N1—C8—C15	176.77 (13)	C6—C7—C9—C10	−43.7 (2)
C1—N1—C8—C7	1.04 (16)	C8—C7—C9—C14	−37.6 (2)
C8—N1—C1—C2	−177.67 (17)	C9—C7—C8—N1	169.40 (13)
C8—N1—C1—C6	−0.58 (17)	N1—C8—C15—N2	150.12 (13)
N3—N2—C15—O1	−7.9 (2)	N1—C8—C15—O1	−30.4 (2)
C15—N2—N3—C16	−171.16 (16)	C7—C8—C15—N2	−35.4 (2)
N3—N2—C15—C8	171.54 (14)	C7—C8—C15—O1	144.08 (15)
N2—N3—C16—C17	179.22 (15)	C10—C9—C14—C13	0.3 (3)
N1—C1—C2—C3	176.36 (17)	C7—C9—C10—C11	173.95 (17)
N1—C1—C6—C7	−0.07 (18)	C14—C9—C10—C11	−1.0 (3)
N1—C1—C6—C5	−175.83 (14)	C7—C9—C14—C13	−174.61 (17)
C2—C1—C6—C7	177.25 (15)	C9—C10—C11—C12	0.8 (3)
C6—C1—C2—C3	−0.3 (3)	C10—C11—C12—C13	0.1 (3)
C2—C1—C6—C5	1.5 (2)	C11—C12—C13—C14	−0.8 (3)
C1—C2—C3—C4	−0.4 (3)	C12—C13—C14—C9	0.6 (3)
C2—C3—C4—F1	−179.10 (17)	N3—C16—C17—C22	16.5 (3)
C2—C3—C4—C5	0.0 (3)	N3—C16—C17—C18	−162.92 (18)
C3—C4—C5—C6	1.2 (3)	C16—C17—C22—C21	−178.75 (17)
F1—C4—C5—C6	−179.77 (15)	C18—C17—C22—C21	0.7 (3)
C4—C5—C6—C1	−1.8 (2)	C16—C17—C18—C19	178.02 (18)
C4—C5—C6—C7	−176.23 (17)	C22—C17—C18—C19	−1.4 (3)
C5—C6—C7—C8	175.53 (17)	C17—C18—C19—C20	1.3 (3)
C5—C6—C7—C9	4.7 (3)	C18—C19—C20—C21	−0.4 (3)
C1—C6—C7—C9	−170.19 (12)	C18—C19—C20—O2	179.97 (18)
C1—C6—C7—C8	0.68 (16)	O2—C20—C21—C22	179.27 (18)
C6—C7—C9—C14	131.12 (17)	C19—C20—C21—C22	−0.4 (3)
C6—C7—C8—C15	−175.85 (15)	C20—C21—C22—C17	0.2 (3)
C8—C7—C9—C10	147.59 (16)		

Symmetry codes: (i) −*x*+2, *y*+1/2, −*z*+3/2; (ii) *x*+1, −*y*+1/2, *z*+1/2; (iii) *x*, *y*−1, *z*; (iv) −*x*+1, −*y*, −*z*+1; (v) −*x*, −*y*+1, −*z*; (vi) *x*, *y*+1, *z*; (vii) −*x*+1, −*y*+1, −*z*+1; (viii) *x*+1, −*y*+3/2, *z*+1/2; (ix) −*x*+1, *y*−1/2, −*z*+1/2; (x) *x*−1, −*y*+3/2, *z*−1/2; (xi) −*x*+2, *y*−1/2, −*z*+3/2; (xii) −*x*+1, *y*+1/2, −*z*+1/2; (xiii) *x*−1, −*y*+1/2, *z*−1/2.

### Hydrogen-bond geometry (Å, °)

**Table:**

Cg1 is the centroid of the N1/C1/C6–C8 ring.

**Table:**

*D*—H···*A*	*D*—H	H···*A*	*D*···*A*	*D*—H···*A*
N1—H1···O1^iv^	0.86	2.03	2.8573 (19)	162
N2—H2A···O1^vi^	0.86	2.32	3.0373 (14)	141
C3—H3···F1^xi^	0.93	2.54	3.473 (2)	177
C16—H16···O1^vi^	0.93	2.50	3.1939 (18)	131
C10—H10···Cg1^vi^	0.93	2.85	3.3736 (17)	117

Symmetry codes: (iv) −*x*+1, −*y*, −*z*+1; (vi) *x*, *y*+1, *z*; (xi) −*x*+2, *y*−1/2, −*z*+3/2.
